# Hydrothermal Synthesis of Silver Vanadium Oxide (Ag_0.35_V_2_O_5_) Nanobelts for Sensing Amines

**DOI:** 10.1186/s11671-015-1119-5

**Published:** 2015-10-21

**Authors:** Haitao Fu, Hui Xie, Xiaohong Yang, Xizhong An, Xuchuan Jiang, Aibing Yu

**Affiliations:** School of Materials and Metallurgy, Northeastern University, Shenyang, 110819 China; Department of Chemical Engineering, Monash University, Clayton, VIC 3800 Australia

**Keywords:** Hydrothermal method, Ag_0.35_V_2_O_5_ nanobelts, Gas sensing, Organic amines, DFT simulation

## Abstract

A simple hydrothermal method for the synthesis of Ag_0.35_V_2_O_5_ nanobelts with the assistance of sodium dodecyl sulfate (SDS) is reported in this study. The experimental variables that may affect the nanoparticle structures were investigated. And several advanced techniques, such as TEM, HRTEM, X-ray diffraction (XRD), were used to characterize the morphology and composition of the as-prepared nanobelts. The mechanism of the formation and growth of Ag_0.35_V_2_O_5_ nanobelts was also investigated and discussed. The results show that SDS, as a weak reducing agent, plays a crucial role in the formation of Ag_0.35_V_2_O_5_. According to N_2_ sorption isothermals, the as-prepared Ag_0.35_V_2_O_5_ nanobelts are found to exhibit relative high surface area. The gas sensing performance of the Ag_0.35_V_2_O_5_ nanobelts towards organic amine was tested. It is found that the nanobelts show superior sensitivity of amine(s) to V_2_O_5_ particles, lower detection limit (5 ppm), and higher selectivity of amine versus ammonia at an optimized working temperature of ~260 °C. Moreover, the density functional theory (DFT) simulation was conducted to better understand the sensing mechanism. These findings may be useful in designing promising materials to detect amine gases for medical or food industrial applications.

**Electronic supplementary material:**

The online version of this article (doi:10.1186/s11671-015-1119-5) contains supplementary material, which is available to authorized users.

## Background

Silver vanadium oxides (SVOs) have attracted increasing attention due to their excellent physicochemical properties and diverse applications in fields of batteries [1], gas sensors [2], surface enhanced Raman spectroscopy (SERS), etc. [3]. Different phases of SVO (e.g., Ag_2_V_4_O_11_ [4–6], AgVO_3_ [7], and Ag_0.33_V_2_O_5_ [8]) have been obtained, depending on different reaction conditions and material stoichiometry [9]. Among them, the type of Ag_x_V_2_O_5_ has been extensively studied recently [9–11], especially applied as battery cathode materials with enhanced cycle performance [8].

The Ag_x_V_2_O_5_ particles are also proposed as a potential candidate for gas sensing because of their high surface-to-volume ratio(s) and unique electronic structure. The investigation of sensing property of this material, however, is little reported. Compared with other types of SVO, Ag_2_V_4_O_11_ and AgVO_3_ have been largely studied as gas sensing materials recently. For example, Mai et al. reported that single β-AgVO_3_ nanowires can be used as a gas sensing material for detecting H_2_S, exhibiting low response concentration of 50 ppm, good selectivity, and short response/recover time of 20 s [2]. Liang et al. demonstrated that Ag_2_V_4_O_11_ nanobelts show a high sensitivity towards ethanol at the working temperature of 200 °C [12]. As one member of the SVO family, therefore, the potential sensing property of Ag_x_V_2_O_5_ is worthy to be further studied.

Organic amines are toxic and widely applied in medicine and food industry. Current techniques in detecting amines are mainly concentrated on liquid chromatogram [13], spectroscopic methods (e.g., fluorescence [14, 15] and optical detection [16]), etc., which may suffer from low efficiency and complicated operation. Resis-chemical sensors are proposed as a good way to overcome these drawbacks, but the sensing materials are vital as a component in the devices. Recently, Ag mesowires and V_2_O_5_ nanowires have been used to detect amines. However, limitations still exist. For example, V_2_O_5_ nanowires show good detection limit (30 ppb) but low sensitivity [17], while Ag mesowires exhibit good sensitivity but undesirable selectivity of amine and ammonia [18]. Here, we propose that the nanostructure of combined vanadium oxide and silver may help to improve the sensing performance.

The functional properties of Ag_x_V_2_O_5_ are heavily dependent on its microstructure and crystalline surface. To will control, hydrothermal method has been widely used. For instance, Liang et al. demonstrated that channel-structured β-Ag_0.33_V_2_O_5_ nanorods can be synthesized by a hydrothermal method at 205 °C for 24 h [11]. Xu et al. reported that Ag_0.33_V_2_O_5_ nanowires with diameter of 80–100 nm and length of several tens of micrometers are prepared at 200 °C for 24 h [8].

In the present work, Ag_0.35_V_2_O_5_ nanobelts, as a potential sensing material, were prepared by a developed hydrothermal method. Various advanced techniques were used for the microstructural characterization, including transmission electron microscopy (TEM) and X-ray diffraction (XRD). The pertinent experimental parameters, such as the type of additives and precursors and ratio of silver to vanadium were investigated. N_2_ sorption isothermals were used to characterize the surface area of the products. The density functional theory (DFT) simulation was conducted to understand gas sensing performance and mechanism in detecting amines.

## Methods

### Preparation of Ag_0.35_V_2_O_5_ Nanobelts

The Ag_0.35_V_2_O_5_ nanobelts were synthesized by a hydrothermal method. Briefly, a mixture of 1.5 mmol vanadium pentoxide powder and 0.6 mmol sodium dodecyl sulfate (SDS; A. R. Grade) was dissolved in 15 ml pure water. Afterwards, various molar ratios of Ag to vanadium were added in the mixture in order to investigate the effect of the ratio on the formation of the nanobelts. To investigate the effect of additives, SDS was replaced with cetyltrimethylammonium bromide (CTAB; A.R. Grade) and/or poly(vinylpyrrolidone) (PVP; *M*_W_ = 55,000). The mixture was then placed into a Teflon-lined stainless steel autoclave. After heating at 180 °C for 24 h, greenish gray precipitates were formed. Finally, the reaction system cooled down to room temperature, and the precipitates were centrifuged, collected, and rinsed by pure water and ethanol several times, and then dried at room temperature for further characterization.

### Characterization

The morphology size and structure of the samples were investigated with a JEOL 1400 microscope (TEM), operated at an accelerated voltage of 100 kV. To characterize the composition of the materials, powder XRD pattern was recorded on a Philip MPD diffractometer with Cu-Kα radiation. The Brunauser-Emmett-Teller (BET) surface area and pore size distribution of the products were obtained from nitrogen physisorption isotherms (adsorption-desorption branches) at 77 K on a Micromeritics ASAP 2020 instrument. Prior to the measurements, the Ag_0.35_V_2_O_5_ and V_2_O_5_ samples were degassed overnight under vacuum at 150 °C to vaporize water molecules adsorbed on the materials.

### Gas Sensing Performance

The gas sensing performance of the materials was conducted by WS-30A gas sensing measurement system. In the test process, the change of the resistance of the sensor in air or in a test gas can be monitored via the voltage (*V*_output_), which is the voltage at the two ends of the reference resistor (*R*_reference_). The sensor was made by dispersing the as-prepared Ag_0.35_V_2_O_5_ nanobelts in tetraethyl ammonium tetrafluoroborate (Sigma-Aldrich, 99 %, as binder) and ethanol to form slurry, then depositing the mixture as a thin film on a clip (3 × 3 mm) with Au electrodes and Pt conducting wires. The gas sensing measurements were carried out at the relative humidity of 30 %.

### Computational Simulations

DFT simulations were used to assist and understand the gas sensing mechanism via the commercial software: Materials Studio (Version 4.3, Accelrys Inc., 2007) with CASTEP Module. The widely used generalized gradient approximation (GGA) with an exchange-correlation functional parameterized by Perdew and Wang (PW91) was employed in this case. All electron calculations and a double numerical basis set with polarization functions (DPN) were employed with a global orbital cut off of 3.7 Å. The total energy convergence was set to be 1 × 10^−6^ Ha [19].

## Results and Discussion

### Synthesis of Ag_0.35_V_2_O_5_ Nanobelts

**Fig. 1:**
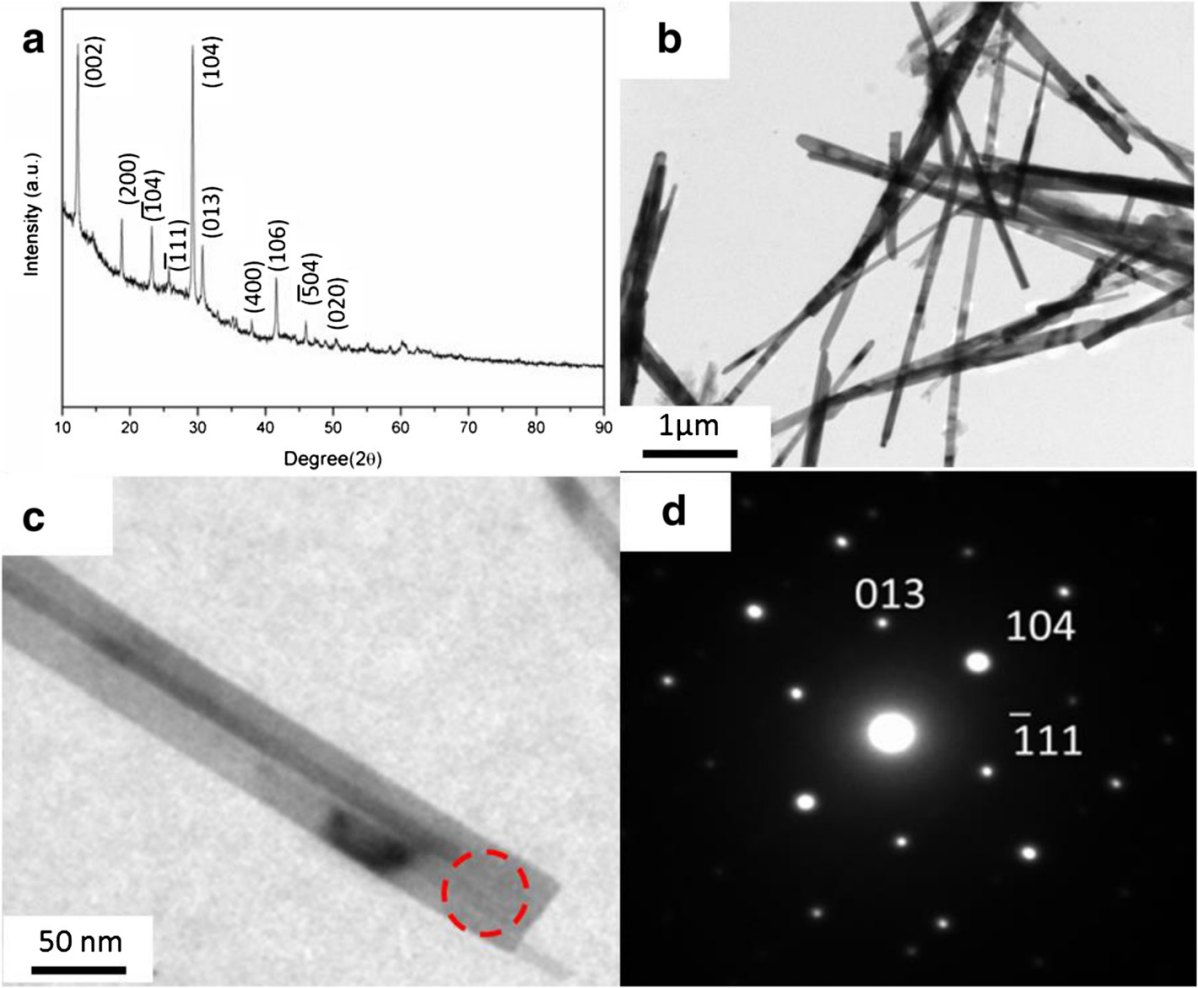
**a** XRD patterns of the nanobelts, showing a typical pattern of Ag_0.35_V_2_O_5_, **b** a TEM image of the Ag_0.35_V_2_O_5_ nanobelts, **c** a TEM image of a single nanobelts, and **d** the corresponding selected area electron diffraction (SAED) pattern

The composition of the product was investigated by XRD technique. Figure 1a shows the XRD pattern. The composition of the sample can be assigned to Ag_0.35_V_2_O_5_, which is prepared by the assistance of SDS at the Ag/V molar ratio of 15:100. The sharp diffraction peaks in the XRD pattern reveal that the as-prepared material is well crystallized. The morphology and size of the nanostructure were investigated by TEM, as shown in Fig. 1b. It is found that the Ag_0.35_V_2_O_5_ is of belt-like structure with width of 50–100 nm and length of 2–5 μm. The selected area electron diffraction (SAED; Fig. 1d) taken from the individual nanowire (Fig. 1c) displays a monoclinic crystalline phase. Three sets of typical crystalline planes can be indexed as {013}, {104}, and {$\overline{1}$11}, respectively, corresponding to the belt-like nanostructures, and the corresponding zone axis is [43$\overline{1}$].

### Effect of Molar Ratios of Ag to Vanadium

**Fig. 2:**
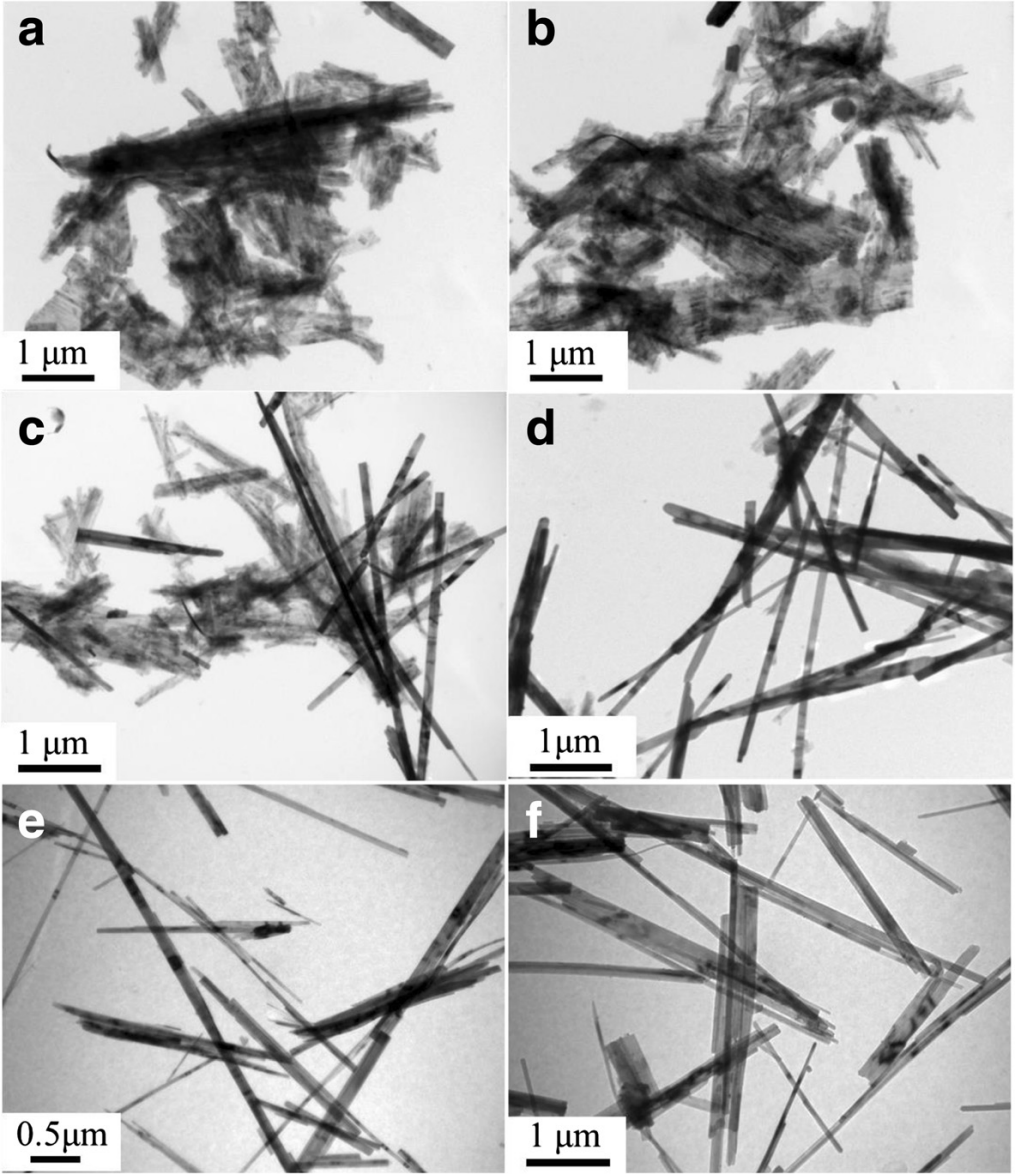
TEM images of the products obtained with different molar ratios of Ag to vanadium: **a** 0, **b** 5, **c** 10, **d** 15, **e** 50, and **f** 100 %

The effect of the different Ag/V molar ratios (0, 5, 10, 15, 50, and 100 %) on the formation of the Ag_0.35_V_2_O_5_ was investigated with the assistance of SDS. The corresponding morphology and size of the materials were observed by TEM, as shown in Fig. 2. It is found that the structure obtained without Ag is the belt-like particles with wide size distribution (width of 0.5–1 μm and length of 1–5 μm), which is more close to 2D structure (Fig. 2a). The morphology of the nanostructure undergoes little change when increasing the Ag/V molar ratio to 5 % (Fig. 2b). Further increasing the molar ratio to 10 %, some uniform nanobelts with ~50 nm in width were formed. At the same time, the wide belts still exist (Fig. 2c). Continuously increasing the ratio of Ag/V to 15 %, most of the products are composed of uniform nanobelts with width of ~50 nm. The morphology and size are similar to those obtained at the ratio of Ag/V of 15 % when the ratio rises to 50 and 100 % (Fig. 2e, f).

**Fig. 3:**
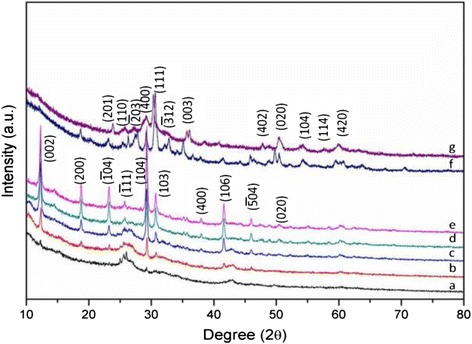
XRD patterns of the nanobelts obtained with different Ag/V molar ratios: (*a*) 0, (*b*) 1, (*c*) 5, (*d*) 10, (*e*) 15, (*f*) 50, and (*g*) 100 %

The composition of the products synthesized with various Ag/V molar ratios was confirmed by XRD, as shown in Fig. 3. Obviously, different Ag/V ratios result in different compositions of the products. It is found that the crystallinity of the nanoparticles increases when increasing the molar Ag/V ratio. When the ratio is lower than 1 %, the product is close to amorphous. When the Ag/V ratio is in the range of 5–15 %, the typical crystalline feature corresponding to Ag_0.35_V_2_O_5_ was observed. Continuously increasing the ratio to 50 %, the composition of the product was identified as Ag_2_V_4_O_11_ but not as Ag_0.35_V_2_O_5_. It means that two types of silver vanadium oxide materials can be prepared by adjusting the Ag/V molar ratios. Therefore, the molar ratio of Ag/V affects not only the morphology but also the composition and crystallinity of the products [6].

### Effect of Additives on the Formation of Ag_0.35_V_2_O_5_ Nanobelts

The effect of vanadium precursors and additives on the formation of the Ag_0.35_V_2_O_5_ nanobelts was studied in this work. The concentration of the additives is kept as 0.6 mmol/L. Sodium vanadate (Na_3_VO_4_ or NaVO_3_), used as precursor in the system, can lead to the formation of other sodium vanadate (e.g., NaV_6_O_15_). To avoid the possible impurity, NH_4_VO_3_ and V_2_O_5_ were selected as the vanadium sources. To control the shape and size, three types of additives were investigated, including SDS, PVP, and CTAB, acting as anionic, neutral, and cationic surfactants with different functions in the formation of silver vanadium oxides. It is noted that, although CTAB can lead to the formation of AgBr precipitate in the presence of Ag^+^, the AgBr, as an intermediate, will decompose eventually and hence Ag nanoparticles can be formed [20, 21].

**Fig. 4:**
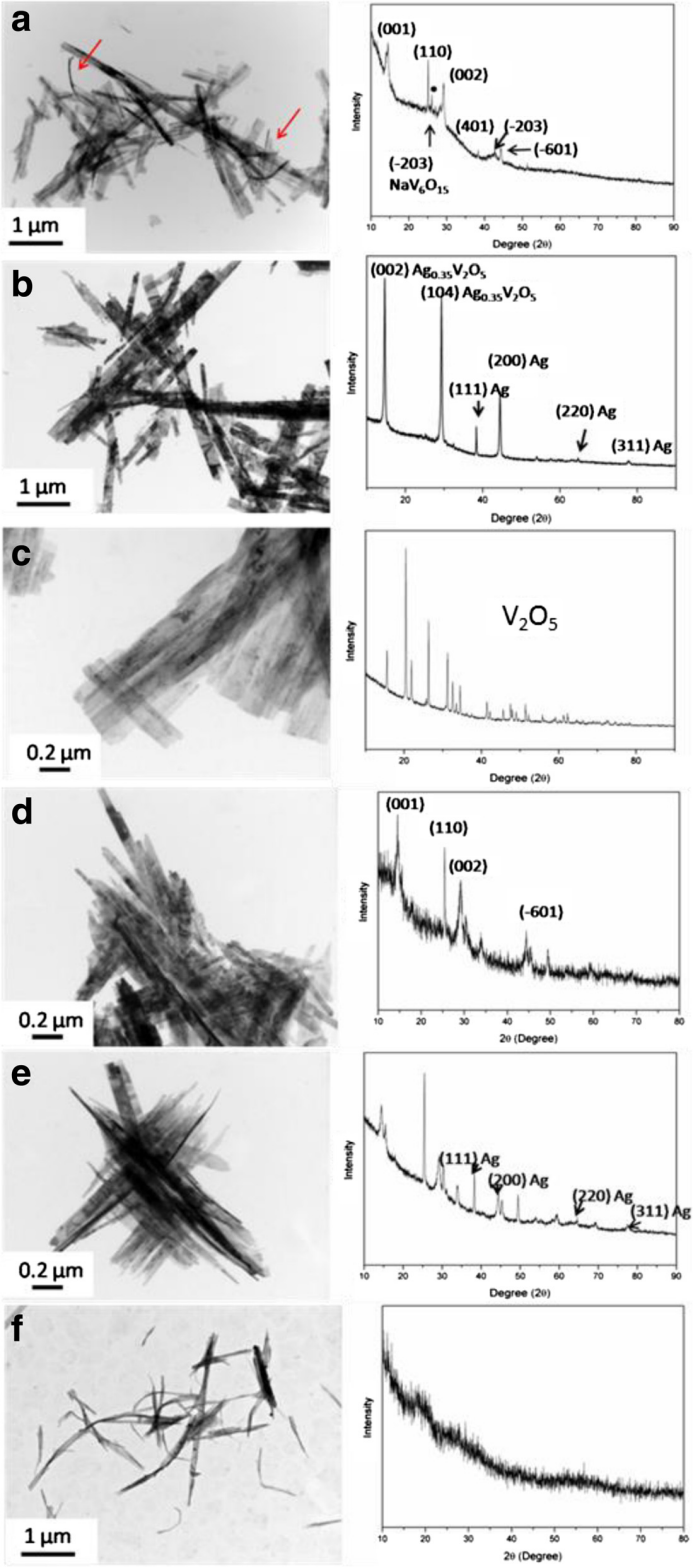
TEM images (*left*) and XRD patterns (*right*) of the products obtained by hydrothermal methods under various reactants. The reactants are as follows: **a** NH_4_VO_3_ + SDS, **b** NH_4_VO_3_ + SDS + Ag (10 %), **c** V_2_O_5_ + Ag (10 %), **d** V_2_O_5_ + PVP, **e** V_2_O_5_ + PVP + Ag (10 %), and **f** V_2_O_5_ + CTAB + Ag (10 %)

The morphology and composition of the samples prepared with different precursors and additives were further identified, as shown in Fig. 4, in which the left column displays the TEM images and the right column shows the corresponding XRD pattern. Figure 4a shows the TEM image and XRD pattern of the product synthesized by NH_4_VO_3_ and SDS, while Fig. 4b shows those obtained by NH_4_VO_3_, SDS, and 10 % (molar ratio) of Ag. In comparison, it is found that the products synthesized with Ag are with higher contrast, suggesting the formation of different nanostructures. The discrepancy in the particle structure is probably attributed to the change of the composition. From XRD patterns, it can be found that the product synthesized without Ag is mainly VO_2_ (B) phase. However, due to the existence of sodium (generating from SDS), an impurity corresponding to NaV_6_O_15_ is formed. According to the XRD pattern shown in Fig. 4b, the products are mainly composed of Ag_0.35_V_2_O_5_ and metallic Ag. That is, these reactants may cause mixed products under such conditions (e.g., Ag particles), and the reason requires further study.

Figure 4c–e shows the products obtained with various sets of reactants of V_2_O_5_ + Ag (10 %, Ag/V molar ratios), V_2_O_5_ + PVP, and V_2_O_5_ + PVP + Ag (10 %). From the TEM images, the belt-like structure is retained in the three products. The XRD result in Fig. 4c indicates that the expected Ag_0.35_V_2_O_5_ cannot be formed without additives (SDS or PVP). But the morphology significantly changes from the original plate-like structure (Additional file 1: Figure S1) to the belt-like layered structure. According to Livage’s study, the formation of vanadium oxides by the hydrothermal method is mainly composed of two processes: hydrolysis and condensation. During these processes, vanadium oxide particles can be restructured, which may explain why the morphology of V_2_O_5_ particles changed in Fig. 4c [22].

Figure 4d shows the morphology and composition of the product produced by V_2_O_5_ and PVP. The corresponding XRD pattern reveals that the nanobelts are VO_2_ (B) phase. With the addition of 10 % Ag^+^, not only V_2_O_5_ but also a small amount of Ag nanoparticles is formed, as shown in Fig. 4e. With the replacement of PVP by CTAB, the belt-like products becomes amorphous, and no Ag and Ag_0.35_V_2_O_5_ are formed, as shown in Fig. 4f. This may be attributed to the formation of amorphous CTAV by the cation [CTA]^+^ (from CTAB) and vanadium, as reported by Luca et al. [23, 24].

On the basis of the above discussion, it can be preliminarily concluded that: (1) Ag_0.35_V_2_O_5_ cannot be prepared without SDS under such conditions; (2) both SDS and PVP can reduce V^5+^ in the absence of Ag^+^, and SDS may introduce a small amount of sodium; (3) the reducing property of PVP is higher than that of SDS, because in the present of Ag^+^, PVP can reduce V_2_O_5_ and Ag^+^ to VO_2_ and Ag, while SDS can partially reduce V_2_O_5_ and Ag^+^ to Ag_0.35_V_2_O_5_; (4) compared to NH_4_VO_3_, V_2_O_5_ as the vanadium precursor can form products of pure of Ag_0.35_V_2_O_5_; and (5) CTAB can lead to an amorphous belt-like nanostructure, instead of Ag_0.35_V_2_O_5_. That is, the appropriate reactants for the synthesis of pure Ag_0.35_V_2_O_5_ are V_2_O_5_, SDS, and Ag^+^, also confirmed by the further investigations with different ratios of SDS to V (5, 10, 20, 30, 40, and 50 %), as shown in Additional file 1: Figure S2.

### Formation Mechanism of Ag_0.35_V_2_O_5_ Nanobelts

The precise formula of Ag_0.35_V_2_O_5_ can be represented as Ag(I)_0.35_V(IV)_0.35_V(V)_1.65_O_5_, which is a non-stoichiometric solid solution of silver in V_2_O_5_ [10]. The formation of Ag_0.35_V_2_O_5_ benefits from the reducing agent which must be weak enough to reduce a small part of V^5+^ to V^4+^ under hydrothermal condition, rather than to fully reduce V^5+^ to form VO_2_. As compared in Fig. 4, only V_2_O_5_ and Ag^+^ cannot lead to the formation of Ag_0.35_V_2_O_5_ (Fig. 4c), while PVP could totally reduce V_2_O_5_ and Ag^+^ to VO_2_ and metallic Ag (Fig. 4d, e). Notably, Ag_0.35_V_2_O_5_ could not be prepared without SDS although some reactants can cause the impurity (Fig. 4a, b). That is, SDS owns a weak reducing property (even weaker than PVP) which is indispensable for the formation of Ag_0.35_V_2_O_5_ at such hydrothermal condition.

**Fig. 5:**
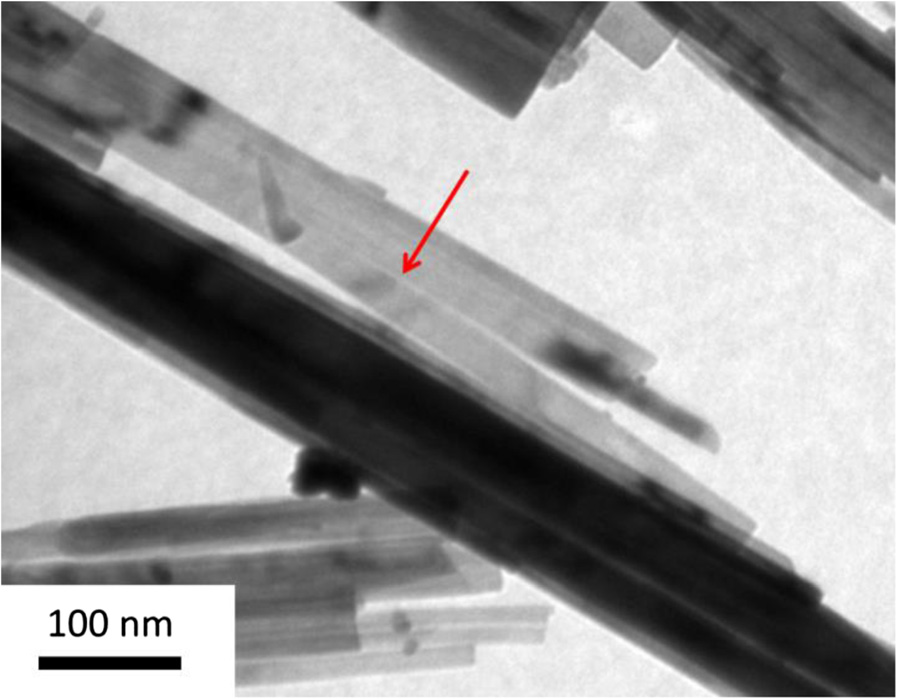
A TEM image of splitting nanobelts

The growth of the nanobelts can be divided into two steps: the formation of layered structure and splitting. The formation of the layered structure can be attributed to its crystal structure. Similar to the structure of Ag_2_V_4_O_11_, VO_6_ octahedral is the basic unit. The octahedral can form the structures of zigzag chains and two-leg ladders which constitute infinite [V_4_O_12_]_n_ quadruple strings. [V_4_O_11_]_n_ layers along the (001) plane are formed by the strings built along *b-axis* and linked by corner-shared oxygen atoms. Intercalation of Ag^+^ between the layers may result in the Ag_0.35_V_2_O_5_ layered structure [8]. However, the layered structure is unstable. A splitting process is then followed, as displayed in Fig. 5. Thus, according to the experimental observation, the formation of layered structures and the splitting process are proposed to elucidate the formation of the nanobelts, in good agreement with the previous study [25].

### Gas Sensing Performance

The gas sensing performance of the as-prepared Ag_0.35_V_2_O_5_ nanobelts was investigated via measuring the selectivity to organic ammines, ammonia, acetone, and alcohols. V_2_O_5_ was used as a comparison material. The morphology is shown in Additional file 1: Figure S1. Both materials are proposed as n-type semiconductors due to their similar crystal structures [11, 26]. To confirm the stability of Ag_0.35_V_2_O_5_, the composition of Ag_0.35_V_2_O_5_ sintered at 400 °C for 10 h in air was measured, as shown in Additional file 1: Figure S3. The results indicate that the Ag_0.35_V_2_O_5_ nanobelts are fairly stable in the sensing environment.

**Fig. 6:**
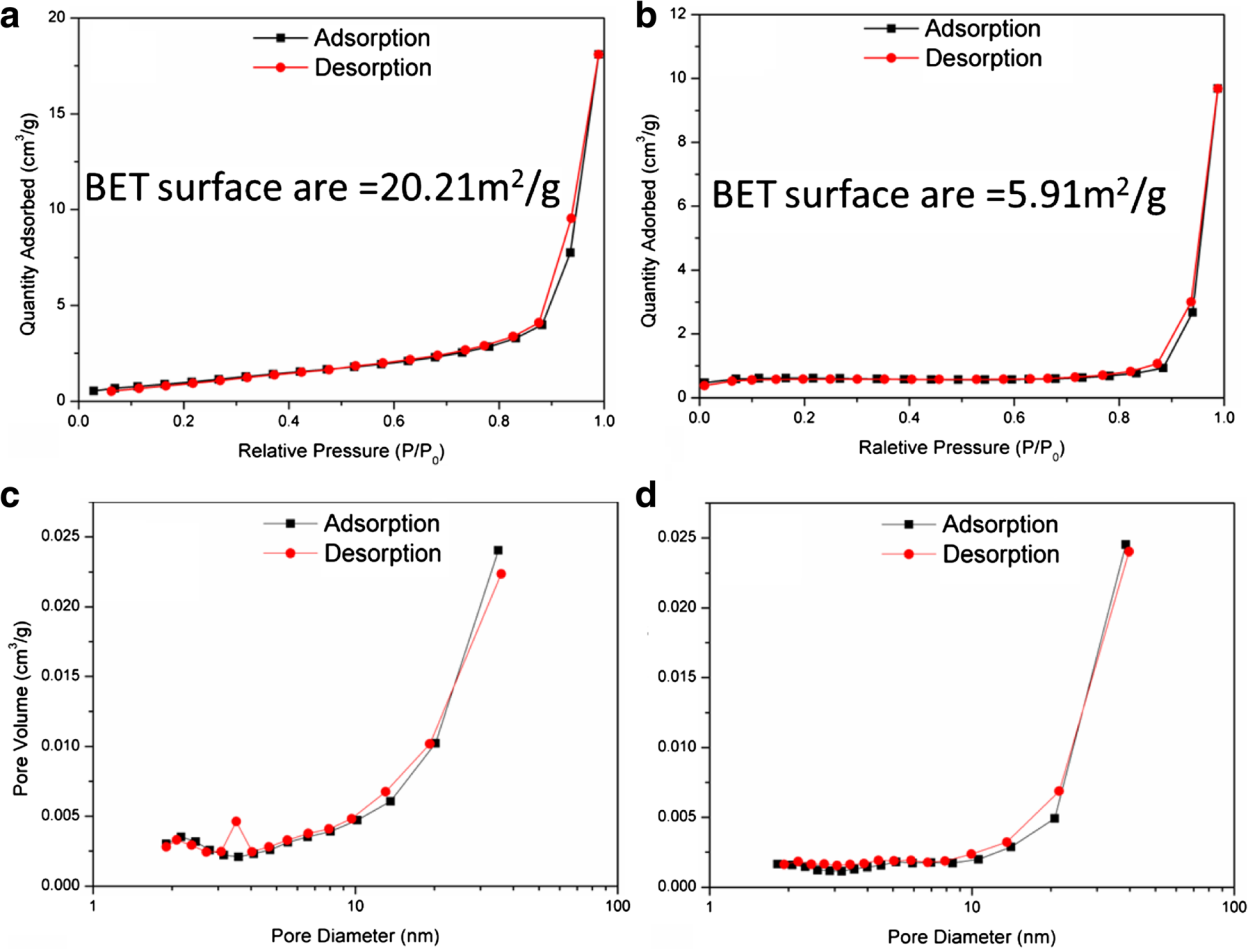
N_2_ sorption isotherms of **a** the Ag_0.35_V_2_O_5_ nanobelts and **b** the V_2_O_5_ particles; the pore size distribution of **c** the Ag_0.35_V_2_O_5_ nanobelts and **d** the V_2_O_5_ particles

Surface area is one of the key factors affecting the gas sensing performance [27]. The surface areas of the as-prepared Ag_0.35_V_2_O_5_ nanobelts and the V_2_O_5_ nanoparticles were measured by the BET method via N_2_ sorption isotherms. The BET surface area of the Ag_0.35_V_2_O_5_ nanobelts is ~20.21 m^2^ g^−1^, higher than the V_2_O_5_ particles (5.91 m^2^ g^−1^), as shown in Fig. 6a, b. This may be the reason that the sensing performance of Ag_0.35_V_2_O_5_ nanobelts would be better than that of V_2_O_5_ particles. The larger surface area of the particles may provide more sites for the adsorption of O_2_ molecules, which plays an important role in the sensing mechanism. The pore size distributions (Fig. 6c, d) derived from both adsorption and desorption branches of the isotherms using the BET method indicate the difference in porosity of the two materials.

**Fig. 7:**
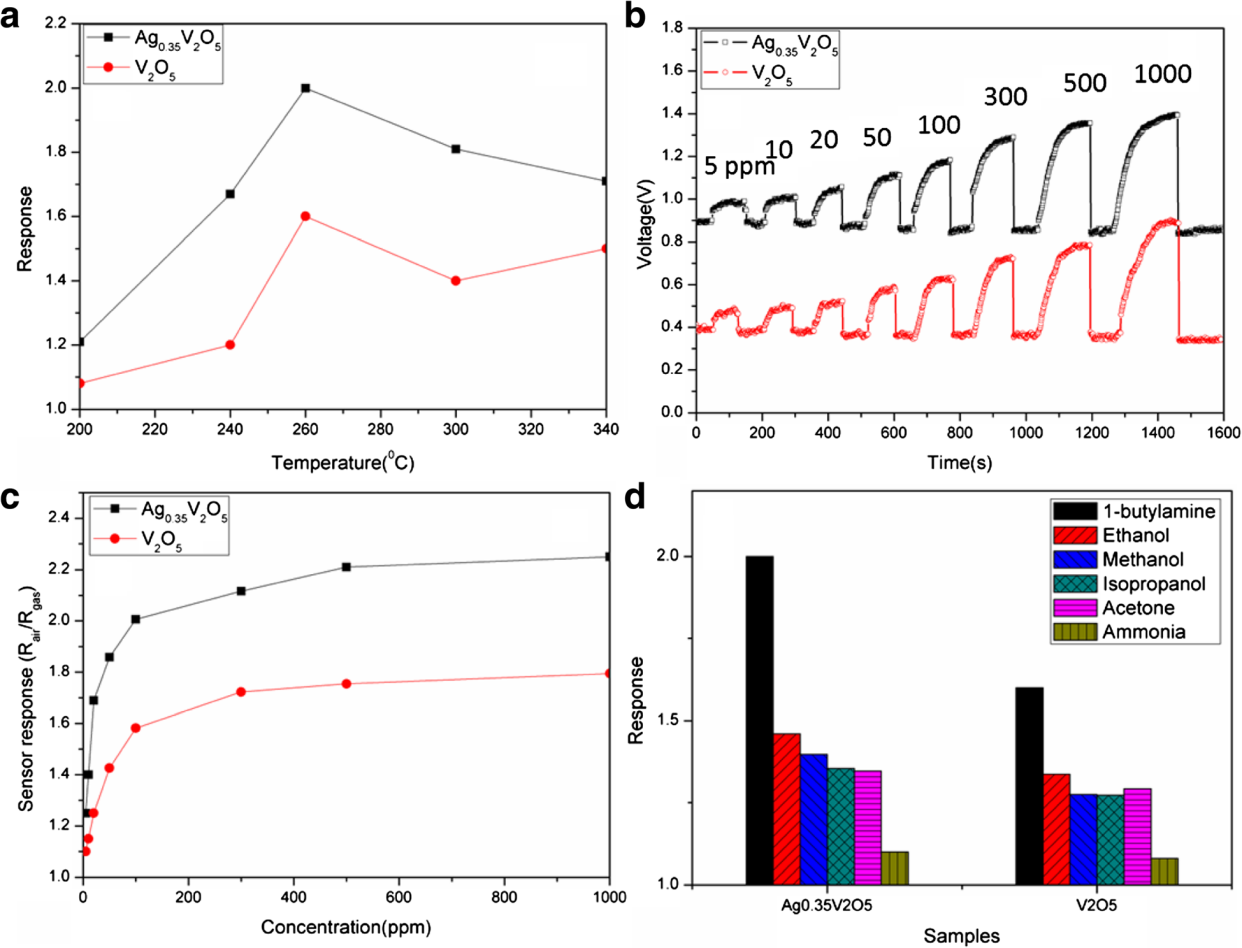
**a** Response to 100 ppm 1-butylamine of the sensors based on Ag_0.35_V_2_O_5_ nanobelts and V_2_O_5_ nanoparticles at various working temperatures. **b** Sensor isothermal response of Ag_0.35_V_2_O_5_ nanobelts to various concentrations of 1-butylamine at the optimized working temperature of 260 °C. **c** Response of the as-prepared Ag_0.35_V_2_O_5_ nanobelts and V_2_O_5_ particles to different concentrations of 1-butylamine testing at the optimized temperature. **d** Relative selectivity of the sensors based on Ag_0.35_V_2_O_5_ nanobelts and V_2_O_5_ nanoparticles to various gases at the optimized working temperature, and the gas concentration is fixed at 100 ppm

Working temperature significantly affects response and further sensitivity [28, 29]. Sensor response (R) is defined as the ratio of the stationary electrical resistance of the sensing materials in the test gas (*R*_gas_) to the resistance in air (*R*_air_), that is, *R* = *R*_air_/*R*_gas_. Figure 7a shows the sensing responses to 100-ppm 1-butylamine of the sensors based on the two materials at different working temperatures. It can be found that the as-prepared Ag_0.35_V_2_O_5_ nanobelts show higher response than V_2_O_5_ particles at the temperature range from 200 to 340 °C, and the optimized working temperatures are both around 260 °C.

Figure 7b shows the typical real-time isothermal response curves of Ag_0.35_V_2_O_5_ nanobelts and V_2_O_5_ particles towards 1-butylamine in the range of 5–1000 ppm at working temperature of 260 °C. It manifests that the response increases with the gas concentration. This sensing material can detect the amine at the low concentration of 5 ppm. The responses of the materials towards 1-butylamine at different concentrations are shown in Fig. 7c, in which the Ag_0.35_V_2_O_5_ nanobelts show higher response than V_2_O_5_ particles, especially at high concentration.

Selectivity is one of the important characteristics of gas sensors, which is proposed to be more important in practical use [30]. Ideally, sensors are expected to exhibit high sensitivity to some gases and low or no sensitivity to others in the same surroundings [31, 32]. The responses of the sensors based on the Ag_0.35_V_2_O_5_ nanobelts and the V_2_O_5_ particles to 100 ppm different gases (e.g., amines, ammonia, acetone, and alcohols) were measured at the working temperature of 260 °C, as displayed in Fig. 7d. For both Ag_0.35_V_2_O_5_ nanobelts and V_2_O_5_ particles, the responses towards the amines are much higher than that of acetone and alcohols, while Ag_0.35_V_2_O_5_ nanobelts show a modest sensitivity to some volatile organic compounds (VOC) (acetone and alcohols). Furthermore, the Ag_0.35_V_2_O_5_ nanobelts exhibit good selectivity to organic amines versus ammonia, suggesting that organic amines can be distinguished from ammonia by this material. The amine-to-ammonia selectivity of Ag_0.35_V_2_O_5_ nanobelts is higher than that of V_2_O_5_ particles.

The response/recovery time (the time required to 90 % of the final equilibrium value) is displayed in Additional file 1: Figure S4. The Ag_0.35_V_2_O_5_ nanobelts show short response/recovery time with less than 50 s from air to 5–100 ppm 1-butylamine at 260 °C, and the recovery time is even shorter (<20 s). Interestingly, the response time increases with gas concentration, which may be due to the low vapor and diffusion rate of 1-butylamine.

**Fig. 8:**
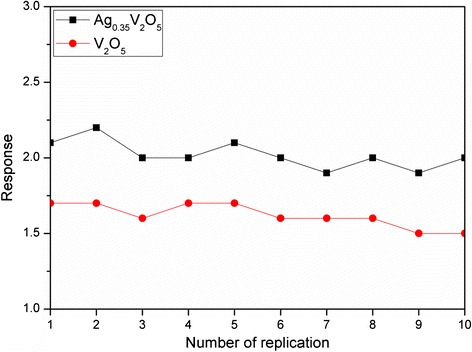
Repeatability tests of two samples (Ag_0.35_V_2_O_5_ nanobelts and V_2_O_5_ particles) towards 100 ppm of 1-butylamine for repeating ten times at 260 °C

The repeatability of the as-prepared samples (Ag_0.35_V_2_O_5_ nanobelts and V_2_O_5_ particles) was also evaluated by testing their response towards 100 ppm of 1-butylamine at 260 °C by repeating ten times. Figure 8 shows that the response of the sensing materials is nearly constant, suggesting a good repeatability for the tested samples under the reported conditions.

### Sensing Mechanism

The gas sensing mechanism for the reducing gases (i.e., ammonia and ethanol) is similar to the previous study [6]. However, the sensing mechanism for organic ammines is slightly different. According to our previous study, the mechanism of SVO-detecting amine was attributed to the intercalation of the layer structure and the interaction between Ag^+^ and amine groups [6]. As the sensor material is exposed to amines, the amine molecules interact with the chemisorbed oxygen species on the surface to form CO_2_, H_2_O, and NO_x_ and then release the trapped electrons and the oxide particles [33]. On the other hand, the style of oxygen ions varies from working temperatures. For example, the stable oxygen ions were O_2_^−^ below 100 °C, O^−^ between 100 and 300 °C, while O^2−^ above 300 °C [34]. Therefore, according to the working temperature in our case (260 °C), this process can be summarized by Eqs. 1, 2, 3:

1 $$ {\mathrm{O}}_{2\left(\mathrm{gas}\right)}\to {\mathrm{O}}_{2\left(\mathrm{ads}\right)} $$

2 $$ {\mathrm{O}}_{2\left(\mathrm{ads}\right)}^{-}+{e}^{-}\to 2{\mathrm{O}}_{\left(\mathrm{ads}\right)}^{-} $$

3 $$ \begin{array}{l}2{\mathrm{C}}_4{\mathrm{H}}_{11}\mathrm{N}{}_{\left(\mathrm{ads}\right)}+\left(2x+27\right){\mathrm{O}}_{\left(\mathrm{ads}\right)}^{-}\to 8{\mathrm{C}\mathrm{O}}_{2\left(\mathrm{gas}\right)}+11{\mathrm{H}}_2{\mathrm{O}}_{\left(\mathrm{gas}\right)}\\ {}+2{\mathrm{NO}}_{\mathrm{x}\left(\mathrm{gas}\right)}+\left(2x+27\right){e}^{-}\end{array} $$

Apart from higher surface area, superior sensing response of Ag_0.35_V_2_O_5_ nanobelts to V_2_O_5_ particles can be attributed to the 1D structure and the unique gas sensing mechanism. It is proposed that one-dimensional structure are expected to significantly enhance performance due to their high surface-to-volume ratio, single crystal, size, and quasi-one-dimensional confinement in nanobelts which is likely to produce a complete depletion of carriers inside [35]. Furthermore, Zhang et al. suggested that the modified ions such as Na^+^ and Ag^+^ could be accommodated on the octahedral sites of the basic structure of metal vanadium oxides, which could further increase the interlayer distance and provide the possibility of accommodating other guest species [19, 36]. On the other hand, the existence of extra Ag^+^ ions may not only lead to the interaction between Ag^+^ and amine groups (−NH_2_) but also result in the presence of lower valence of vanadium (V^4+^). The lower valence can further increase the mobility of main carries and enhance the change of electrical conductivity and sensitivity [6, 19]. In addition, the higher sensitivity of Ag_0.35_V_2_O_5_ than V_2_O_5_ may be also attributed to the decreased electronegativity of surface V in Ag_0.35_V_2_O_5_, resulting in adsorbing more active groups of amines on the surface of Ag_0.35_V_2_O_5_. This means a deeper space charge layer will be created. More electrons can be released and a higher conductivity change is achieved when the target gas molecules react with the adsorbed oxygen species [19]. As a result, the Ag_0.35_V_2_O_5_ nanobelts exhibit higher sensitivity than V_2_O_5_.

For better understanding different responses of alcohols and amine, DFT simulation was performed to calculate the interaction of the adsorbed gas molecules. Generally, the diffusion rate of molecules decreases with the increase of their molecular weights. The calculated distance from O to nearest C for methanol, ethanol, and isopropanol are 1.431, 1.435, and 1.441 Å, respectively, which are in good agreement with reported values [19]. The distance from N to nearest C for 1-butylamine is 1.470 Å, which means C–N bond in the amine is easier to break down.

**Fig. 9:**
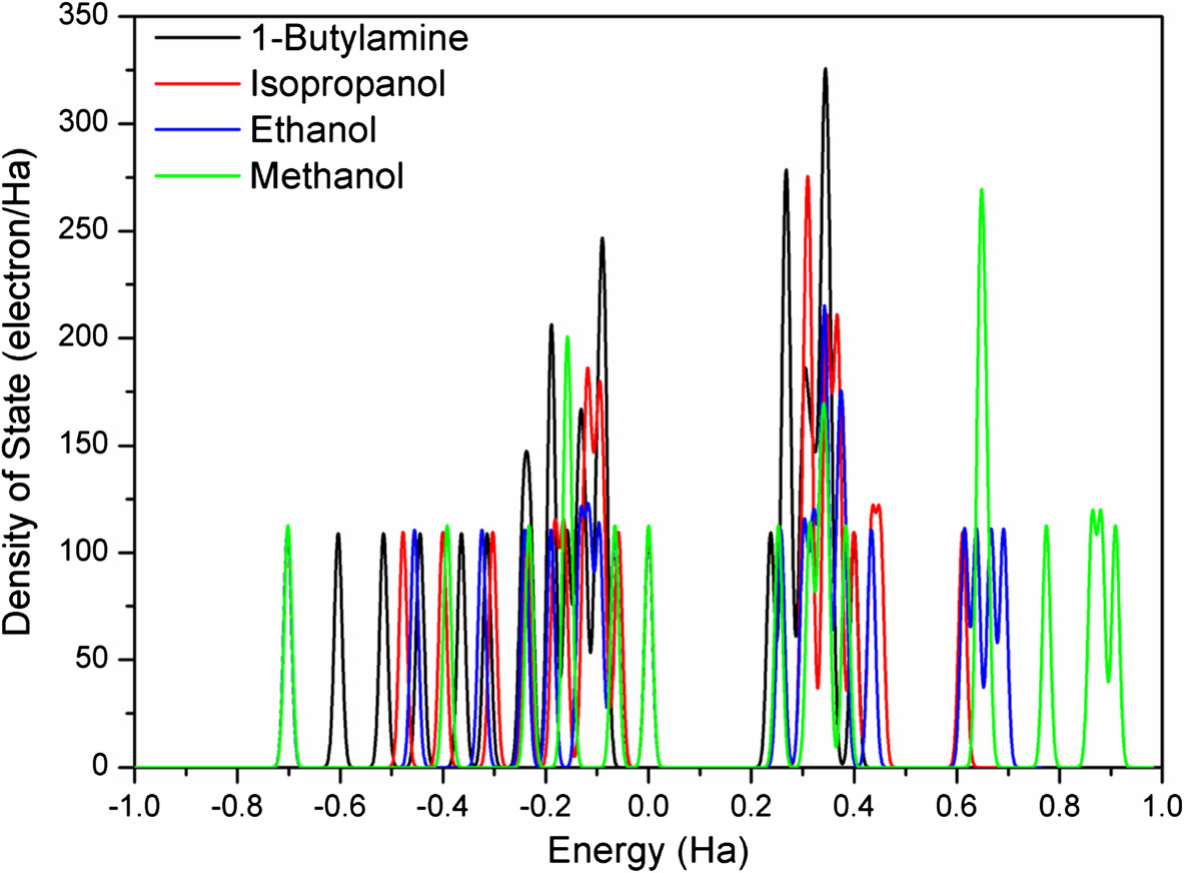
Total DOS plots of methanol, ethanol, isopropanol, and 1-butylamine

Furthermore, the electrostatic interaction between the oxygen species and the gas molecule is one of the important factors that influence the gas sensing performance. The calculated Mulliken charges of O in the three alcohols (methanol, ethanol, and isopropanol) are of −0.506, −0.492, and −0.484 e, respectively, and that of N in 1-butylamine is of 0.448 e. The repulsion interaction between the molecule and the oxygen species becomes weak with the increase of the number of carbon chains, especially for the amine with low Mulliken charge. Thus, more amine molecules can be absorbed on the surface. Therefore, the highest response was obtained from detecting 1-butylamine. It is noted that isopropanol with the longest O–C bond and the lowest Mulliken charge among the alcohols shows lower response than ethanol, which may be attributed to steric hindrance. The density of states (DOS) of the three alcohols and the amine were shown in Fig. 9, suggesting that 1-butylamine molecules are more localized at the bottom of the conduction band, which means it is easier to hybridize with the metal oxide *d* orbital. Further studies, such as the effect of viscosity, interaction between surface and gas molecules, and diffusion rate of various gas species on the surface/interface of the nanostructures will be conducted in the future. The acetone molecule with lower O Mulliken charge (−0.375 e) exhibited lower response than 1-butylamine, probably due to the structure difference.

## Conclusions

We have demonstrated a facile hydrothermal approach to generate Ag_0.35_V_2_O_5_ nanobelts with the assistance of SDS under mild condition. The formation and growth of such 1D nanoparticles under various experimental parameters have been investigated and analyzed. Compared to CTAB and PVP, SDS is proposed to act as a weak reducing agent and plays a key role in the formation of Ag_0.35_V_2_O_5_ nanobelts. The gas sensing performance for this material has been conducted. Compared with naked V_2_O_5_ particles, the as-prepared Ag_0.35_V_2_O_5_ nanobelts exhibit higher sensing response towards amines (e.g., 1-butylamine, *R*_1-butylamine_ = 2.2, while *R*_V2O5_ = 1.6, at 100 ppm), low detection limit (5 ppm), and high selectivity of organic amines versus ammonia. DFT simulation has been used to analyze the structure of the target gas molecules for better understanding of the sensing mechanism. The calculation indicates that the Mulliken charge of N in 1-butylamine is much smaller than those of alcohols, which means the repulsion interaction between the amine molecules and the oxygen species is weaker than alcohol molecules. More 1-butylamine molecules are adsorbed on the surface. And it is easier for N–C bond to break down due to the longer bond length. This study may be useful for the practical use of the SVO-based material in medical and food industry.

## Additional file

Additional file 1:
**The TEM image of V**_**2**_ **O**_**5**_ **as a reactant is displayed in Fig. S1.** The effects of SDS/V molar ratio on the morphology of the as-prepared nanobelts is shown in **Fig. S2**. **Fig. S3** shows the stability of Ag_0.35_V_2_O_5_ nanostructure by calcining the material at 400 °C for 10 hours in air. **Fig. S4** shows the response/revovery time for this material. The details can be seen in Supporting Information.
