# Supplementary material for: Hydrothermal Synthesis of Silver Vanadium Oxide (Ag0.35V2O5) Nanobelts for Sensing Amines
Source: Nanoscale Res Lett. 2015 Oct 21;10:411. doi: 10.1186/s11671-015-1119-5 (PMC4614852; doi:10.1186/s11671-015-1119-5)
Supplement: Additional file 1: — The TEM image of V 2 O 5 as a reactant is displayed in Fig. S1. The effects of SDS/V molar ratio on the morphology of the as-prepared nanobelts is shown in Fig. S2. Fig. S3 shows the stability of Ag0.35V2O5 nanostructure by calcining the material at 400 °C for 10 hours in air. Fig. S4 shows the response/revovery time for this material. The details can be seen in Supporting Information. [file 11671_2015_1119_MOESM1_ESM.doc]

*Supporting Information*

Hydrothermal Synthesis of Silver Vanadium Oxide Nanobelts (Ag_0.35_V_2_O_5_) for Sensing Amines

Haitao Fu,^1^ Xiaohong Yang,^1^^[[1]](#footnote-1)^* Hui Xie,^1^ Xizhong An,^1**^ Xuchuan Jiang,^2^Aibing Yu^2^

^1^ *School of Materials and Metallurgy, Northeastern University, Shenyang 110819, China*

*^2^ Department of Chemical Engineering, Monash University, Melbourne 3800, Australia*


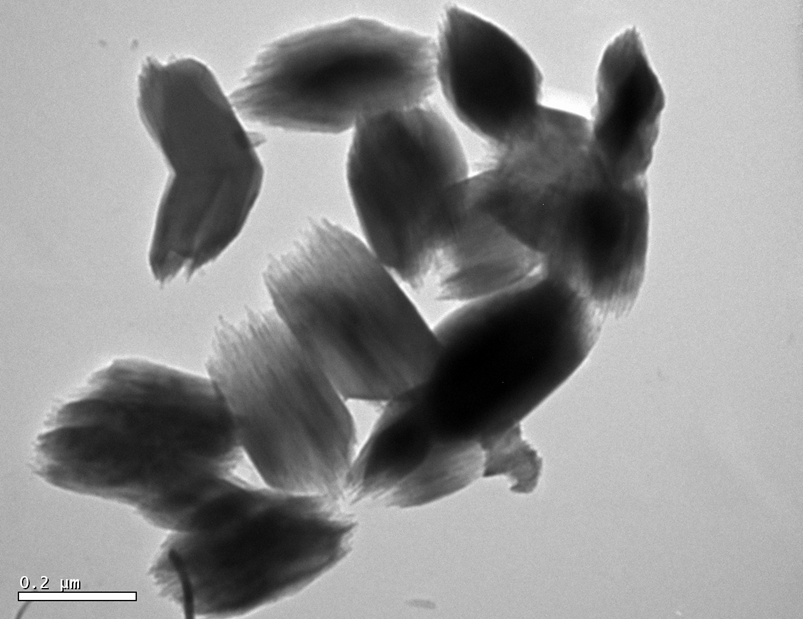


Fig.S1 A TEM image of the V_2_O_5_ reactant.


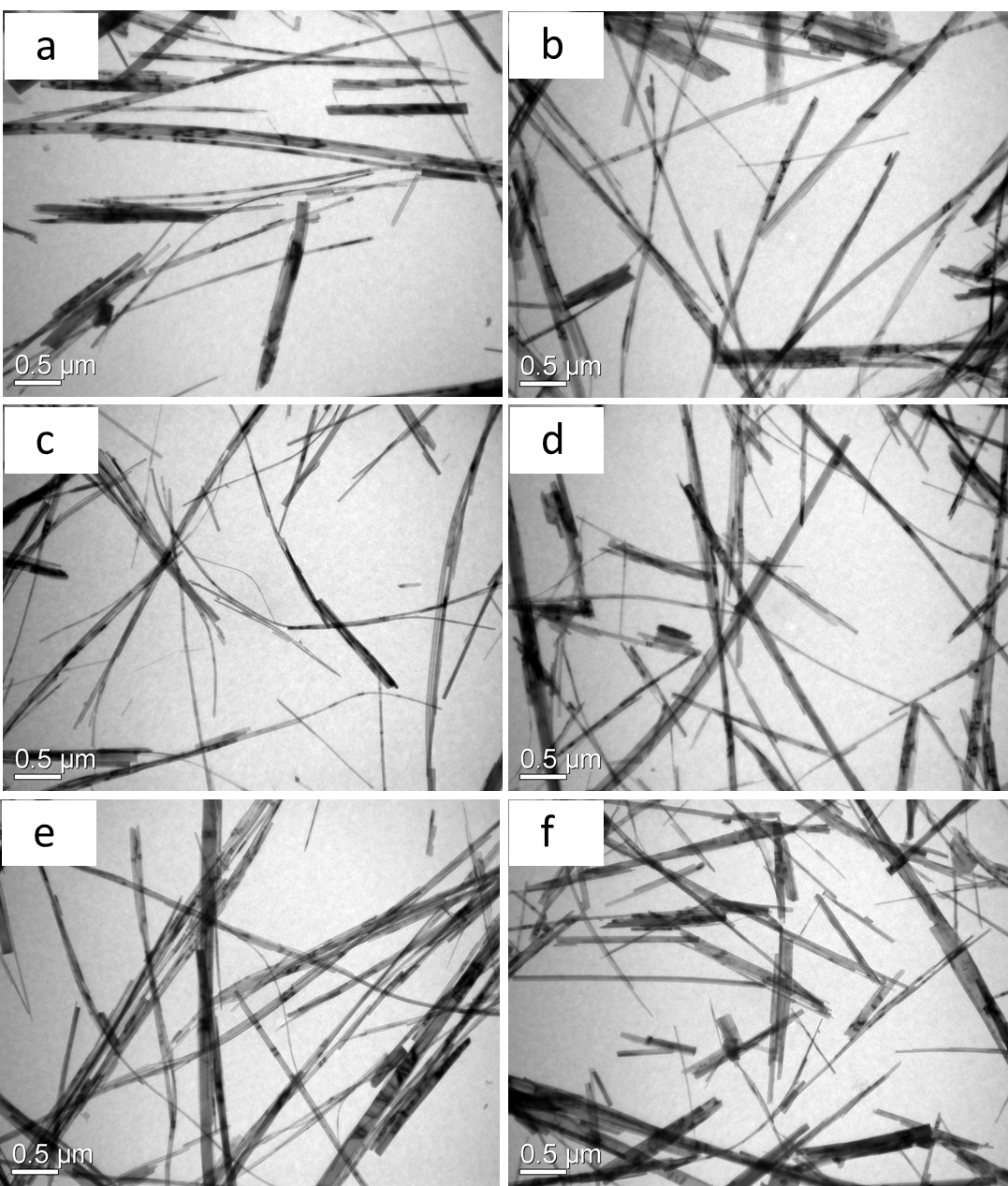


Fig.S2 TEM images of the Ag_0.35_V_2_O_5_ nanobelts at the molar ratio of SDS to V of (a) 5%, (b) 10%, (c) 20%, (d) 30%, (e) 40%, and (f) 50%.


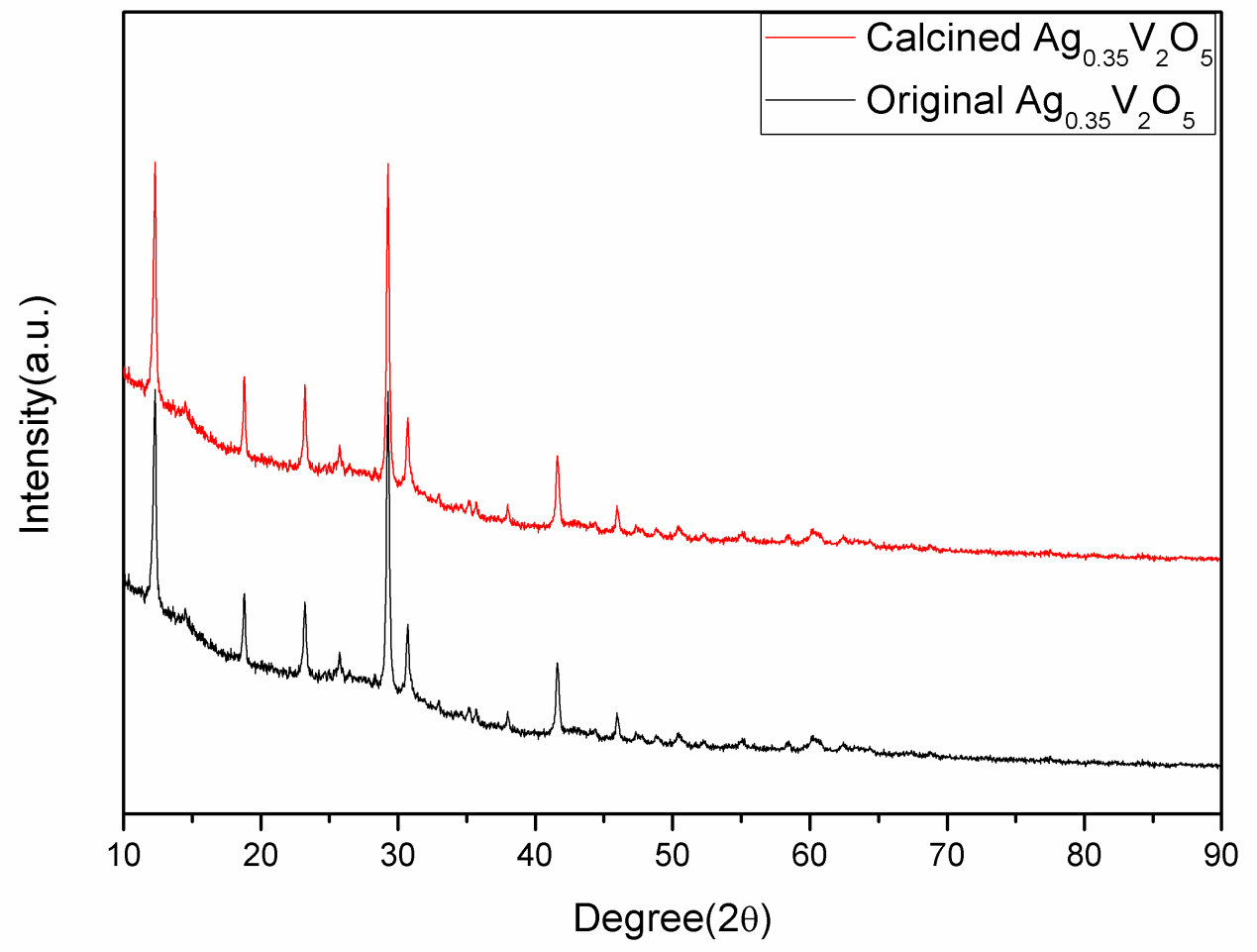


Fig.S3 XRD patterns of the original Ag_0.35_V_2_O_5_ nanobelts and calcined Ag_0.35_V_2_O_5_ nanobelts at 400°C for 10 hours in air.


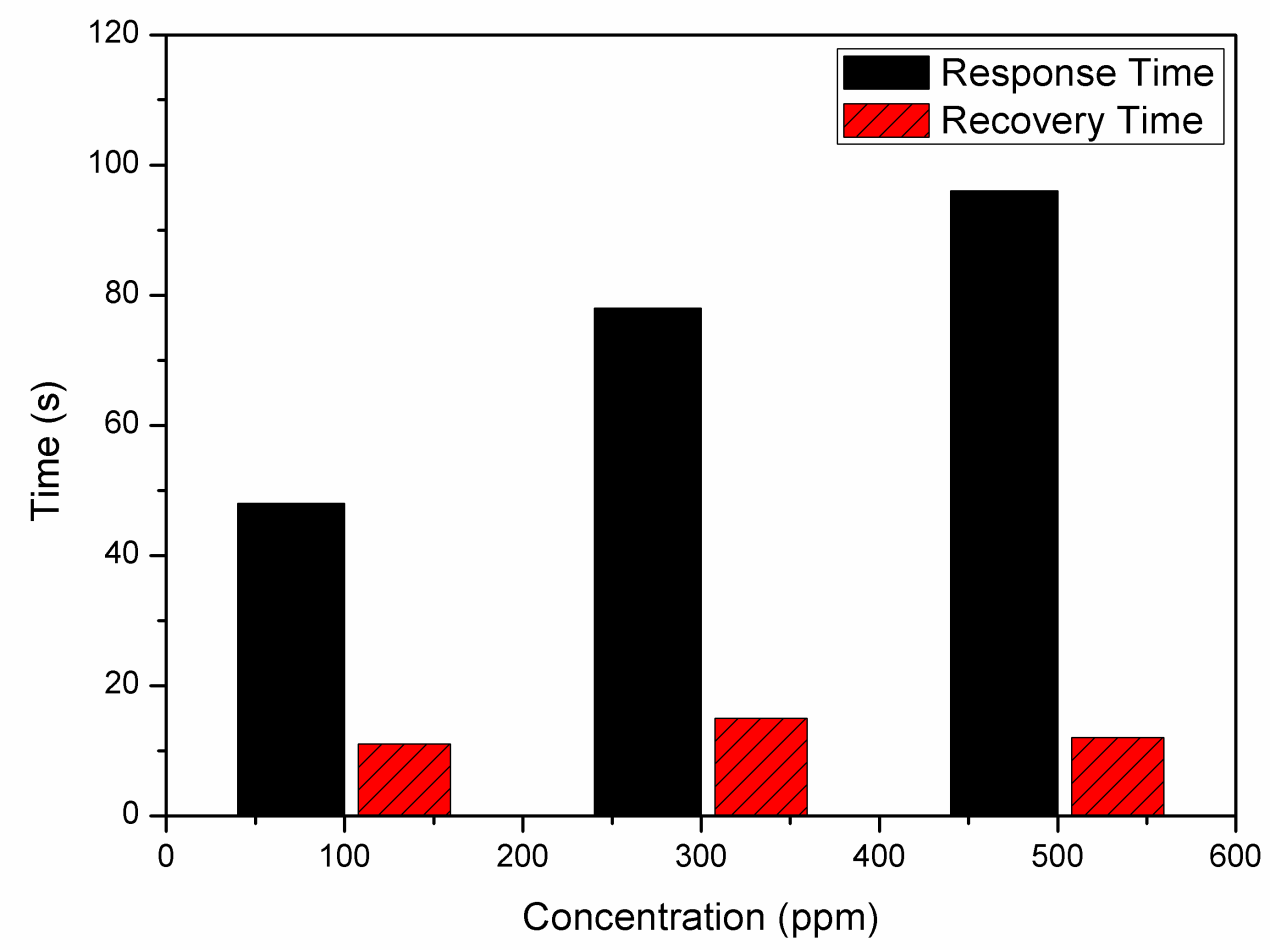


Fig. S4 The response/recovery time with various concentrations.

1. * Correspondence: [yangxh@smm.neu.edu.cn](mailto:yangxh@smm.neu.edu.cn)., [anxz@mail.neu.edu.cn](mailto:anxz@mail.neu.edu.cn) [↑](#footnote-ref-1)
